# Gallic Acid–Triethylene Glycol Aptadendrimers Synthesis, Biophysical Characterization and Cellular Evaluation

**DOI:** 10.3390/pharmaceutics14112456

**Published:** 2022-11-14

**Authors:** André Miranda, Roi Lopez-Blanco, Jéssica Lopes-Nunes, Ana M. Melo, Maria Paula Cabral Campello, António Paulo, Maria Cristina Oliveira, Jean-Louis Mergny, Paula A. Oliveira, Eduardo Fernandez-Megia, Carla Cruz

**Affiliations:** 1 CICS-UBI—Centro de Investigação em Ciências da Saúde, Universidade da Beira Interior, Av. Infante D. Henrique, 6201-506 Covilhã, Portugal; 2 Centro Singular de Investigación en Química Biolóxica e Materiais Moleculares (CIQUS), Departamento de Química Orgánica, Universidade de Santiago de Compostela, Jenaro de la Fuente s/n, 15782 Santiago de Compostela, Spain; 3 iBB—Institute for Bioengineering and Biosciences, Instituto Superior Técnico, Universidade de Lisboa, Av. Rovisco Pais, 1049-001 Lisboa, Portugal; 4 Associate Laboratory i4HB—Institute for Health and Bioeconomy at Instituto Superior Técnico, Universidade de Lisboa, Av. Rovisco Pais, 1049-001 Lisboa, Portugal; 5 Centro de Ciências e Tecnologias Nucleares, Instituto Superior Técnico, Universidade de Lisboa, Estrada Nacional 10 (km 139.7), 2695-066 Bobadela, Portugal; 6 Departamento de Engenharia e Ciências Nucleares, Instituto Superior Técnico, Universidade de Lisboa, Estrada Nacional 10 (km 139.7), 2695-066 Bobadela, Portugal; 7 Laboratoire d’Optique et Biosciences, Ecole Polytechnique, CNRS, INSERM, Institut Polytechnique de Paris, 91128 Palaiseau, France; 8 Centre for Research and Technology of Agro-Environmental and Biological Sciences (CITAB), Inov4Agro, University of Trás-os-Montes and Alto Douro (UTAD), Quinta de Prados, 5000-801 Vila Real, Portugal; 9 Departamento de Química, Universidade da Beira Interior, Rua Marquês de Ávila e Bolama, 6201-001 Covilhã, Portugal

**Keywords:** gallic acid–triethylene glycol dendrimers, G-quadruplex aptamers, acridine orange ligands, aptadendrimer, biophysical studies

## Abstract

Herein, we describe the synthesis of an aptadendrimer by covalent bioconjugation of a gallic acid–triethylene glycol (GATG) dendrimer with the G-quadruplex (G4) AT11 aptamer (a modified version of AS1411) at the surface. We evaluated the loading and interaction of an acridine orange ligand, termed C_8,_ that acts as an anticancer drug and binder/stabilizer of the G4 structure of AT11. Dynamic light scattering experiments demonstrated that the aptadendrimer was approximately 3.1 nm in diameter. Both steady-state and time-resolved fluorescence anisotropy evidenced the interaction between the aptadendrimer and C_8_. Additionally, we demonstrated that the iodine atom of the C_8_ ligand acts as an effective intramolecular quencher in solution, while upon complexation with the aptadendrimer, it adopts a more extended conformation. Docking studies support this conclusion. Release experiments show a delivery of C_8_ after 4 h. The aptadendrimers tend to localize in the cytoplasm of various cell lines studied as demonstrated by confocal microscopy. The internalization of the aptadendrimers is not nucleolin-mediated or by passive diffusion, but via endocytosis. MTT studies with prostate cancer cells and non-malignant cells evidenced high cytotoxicity mainly due to the C_8_ ligand. The rapid internalization of the aptadendrimers and the fluorescence properties make them attractive for the development of potential nanocarriers.

## 1. Introduction

Cancer is a major public health problem that, unfortunately, is growing worldwide. Prostate cancer, like any other cancer, is a cellular disease caused by the alteration of specific genes or changes in cellular signals that lead to abnormal cell growth and proliferation maturation [1]. Chemotherapy, radiotherapy, and surgery are the preferred therapies; however, off-target activity/reactivity and toxicity are major shortcomings [2]. One of the best ways to address these weaknesses is using drug delivery systems that enhance the tumor targeting, tumor uptake, and internalization of the drug by the tumor cells. Several nanostructures, such as dendrimers, have been used to deliver drugs [3]. Dendrimers are synthetic tree-like macromolecules composed of repetitive layers of branching units that are prepared in a controlled iterative process, through generations with discrete properties [4]. As opposed to classical linear polymers, dendrimers are monodispersed and globular [5]. Their quantized nature allows a degree of control over properties/applications unattainable by linear polymers, which has been coined as the “dendritic effect”. In addition, their hydrophobic–hydrophilic character is intrinsically related to the anionic, neutral, or cationic terminal functionalities on the surface [6]. Aptamers have been widely used as promising targeting moieties due to hallmark properties, such as low immunogenicity, easy synthesis, and high specific binding affinity [7]. They can be used to modify drug-loaded nanocarriers for targeted drug delivery. For example, AS1411 is an aptamer formed by a G-rich sequence that can fold into a specific secondary structure, more specifically into a G-quadruplex (G4) conformation [8,9]. The nucleolin (NCL) protein, a tumor biomarker overexpressed in the cancer cell surface, is recognized by AS1411 and its derivatives with high affinity. After binding to NCL, AS1411 can interfere with its oncogenic functions [10,11]. AS1411 has demonstrated an excellent safety profile and ability to induce durable responses in some patients with intractable cancers. Unfortunately, the suboptimal pharmacology and low potency of AS1411 may limit its future development as it is rapidly cleared out from the body [10]. More promising alternatives to AS1411 include versions with modified nucleobases or backbones (such as AT11, AT11-L0, and AT11-B0) or with modifications at the terminus of sequences (such as AS1411-N6) [12,13] to be used as promising drug delivery platforms due to their safety profile, ability to induce durable responses, and higher drug accumulation in some cancer cells [8,9] due to NCL targeting [14].

**Figure 1:**
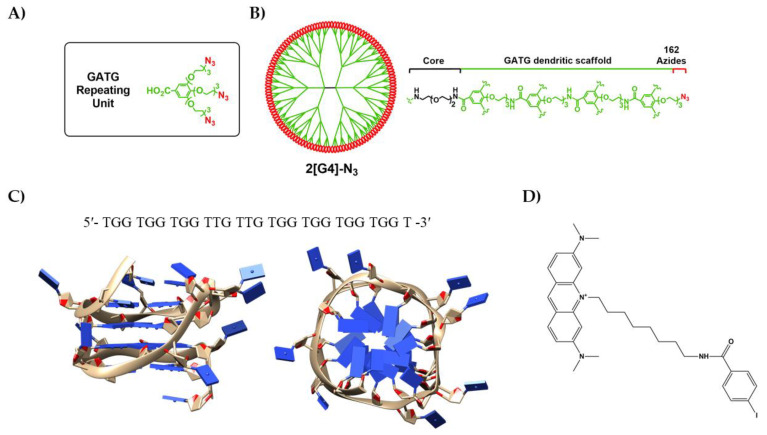
(**A**) Chemical structure of the GATG repeating unit. (**B**) 2[G4]-N_3_ dendrimer with 162 terminal azides. (**C**) G-rich nucleotide sequence and tridimensional structure of AT11 aptamer (PDB entry 2N3M) and (**D**) chemical structure of 10-(8-(4-iodobenzamide)octyl))-3,6-bis(dimethylamine) acridinium iodide, also known as C_8_.

In the current study, we describe an aptamer–dendrimer conjugate (aptadendrimer) composed of a gallic acid–triethylene glycol (GATG) dendrimer (Figure 1A,B) of fourth generation [15] functionalized with the AT11 aptamer, a modified version of AS1411, in which one thymine was removed from the bulge (Figure 1C) [16,17]. We have studied the loading of this aptadendrimer with an acridine orange ligand, termed C_8_, that acts as an anticancer drug and binder/stabilizer of the G4 structure of AT11 (Figure 1D). The aptadendrimer conjugate with C_8_ was characterized in terms of size, charge, and drug release. The cell viability, uptake, and delivery were evaluated into prostate cancer and healthy cell lines. Altogether, our work describes and opens the way for the design and synthesis of aptadendrimers with potential as effective drug delivery systems for cancer therapeutic strategies.

## 2. Materials and Methods

### 2.1. Oligonucleotides and Reagents

AT11 aptamer (5′-TGGTGGTGGTTGTTGTGGTGGTGGTGGT-3′) and AT11-NH_2_ modified oligo were purchased lyophilized with double-HPLC grade purification from Eurogentec (Liège, Belgium). The oligonucleotide concentration was determined using the molar extinction coefficient (ε) provided by the manufacturer and the absorbance at 260 nm. Oligonucleotides were prepared in the buffer according to previously optimized conditions (20 mM phosphate buffer pH = 6.9 supplemented with 65 mM KCl) [18]. Solutions were prepared in ultrapure water (18.2 Ω cm^−1^ resistivity), purified with a milli-Q system from Millipore (Burlington, MA, USA). All chemicals were purchased from Sigma-Aldrich (St. Louis, MI, USA) or Fluka, unless otherwise noted. All solvents were HPLC grade, purchased from Scharlab, Sigma-Aldrich, or Acros Organics. Et_3_N and DMSO were dried under 4Å molecular sieves. DBCO-PEG_13_-NHS ester was purchased from Tebu-bio (Le Perray-en-Yvelines, France). Cy5-DBCO was purchased from Lumiprobe (Hannover, Germany). Amicon purifications were performed on Amicon Ultra-15 Centrifugal Filter Unit MWCO 3 kDa (Sigma-Aldrich, USA). The acridine orange derivative 10-(8-(4-iodobenzamide)octyl))-3,6-bis(dimethylamine) acridinium iodide (C_8_) was synthesized as described elsewhere [19] and dissolved in DMSO at a final concentration of 10 mM. Subsequent dilutions were made with working buffer or cell culture medium. The cells PC-3 (CRL-1435™) and DU-145 (HTB-81™) were purchased from the American Type Culture Collection (ATCC, Manassas, VA, USA) and PNT1A (catalogue number 95012614) were purchased from the European Collection of Cell Cultures (ECACC, Wiltshire, UK).

### 2.2. Synthesis of 2[G4]-N_3_ Dendrimer

The repeating unit of GATG dendrimers (Figure 1A) was synthesized according to [20] using an efficient, safe (green chemistry principles), and cost-effective synthetic route of 4 steps. This repeating unit was employed for the divergent synthesis of dendrimers as previously reported [21]. Briefly, the GATG repeating unit was firstly treated with hydroxybenzotriazole (HOBt) and 1-ethyl-3-(3-dimethylaminopropyl)carbodiimide (EDC) to promote the coupling of two repeating units to a 2,2′-(ethylenedioxy)bis(ethylamine) linker and produce 2[G1]-N_3_, a dendrimer of first generation with 6 terminal azides. After the reduction of the azides with triphenylphosphine (Ph_3_P), via the Staudinger reaction, the resulting terminal amines were again reacted with a second layer of repeating units, and these steps were repeated until the dendrimer achieved the fourth-generation 2[G4]-N_3_ with 162 terminal azides (Figure 1B).

### 2.3. Aptadendrimer Synthesis: Surface Functionalization of 2[G4]-N_3_ with AT11 Aptamer and Fluorophore Labelling

**Figure 2:**
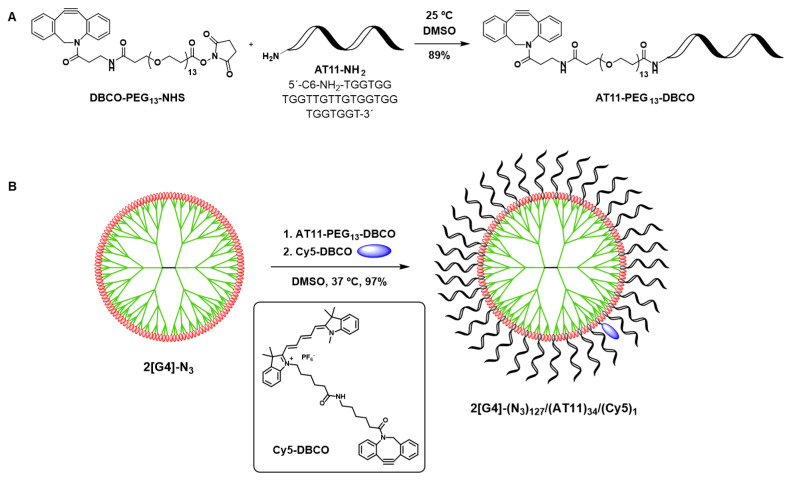
Surface functionalization of 2[G4]-N_3_ dendrimer with AT11 aptamer and fluorophore labelling. (**A**) Functionalization of AT11 with a DBCO cyclooctyne and (**B**) AT11 bioconjugation on dendrimer surface by SPAAC.

The bioconjugation process of the aptamer to the 2[G4]-N_3_ dendrimer was carried out in two phases. Both steps are described in detail in the Supporting Information. First, the AT11 aptamer, modified with an NH_2_ group, was functionalized with a DBCO-PEG_13_-NHS ester (Figure 2A). The long PEG_13_ linker was selected to increase the distance between the dendrimer and the aptamer and, thus, to reduce possible electrostatic repulsions between aptamers. The dibenzocyclooctyne (DBCO) was chosen as a chemical handle for the subsequent bioconjugation step between the dendrimer and AT11-PEG_13_-DBCO by means of a strain-promoted azide–alkyne cycloaddition (SPAAC) [22,23,24] (Figure 2B). The reaction progress of the SPAAC was monitored by polyacrylamide gel electrophoresis (PAGE). Finally, the aptadendrimer was labelled with a Cy5-DBCO fluorophore to allow its visualization in confocal microscopy experiments. (Figure 2B).

### 2.4. Aptadendrimer Characterization

Dynamic light scattering (DLS) measurements were performed on a Malvern Nano ZS (Malvern Instruments, Malvern, UK), operating at 633 nm with a 173° scattering angle, at 25 °C. DLS mean diameters were obtained from the volume particle size distribution provided by the Malvern Zetasizer Software. DLS histograms were obtained from the volume particle size distributions. Z-potential values were obtained by laser Doppler anemometry (LDA), measuring the mean electrophoretic mobility (Malvern Zetasizer Nano ZS, Malvern Instruments).

UV-Vis spectra were recorded on a Jasco V-630 (Tokyo, Japan) and circular dichroism (CD) measurements on a Jasco J-720 (Tokyo, Japan; 100 nm/min of scanning speed ranging from 200 to 400 nm and signal-averaged over three scans). Fluorescence spectra were recorded using a quartz suprasil cuvette (Hellma, Müllheim, Germany) on a Horiba Jobin-Yvon Fluoromax-3 (Tokyo, Japan) coupled to a Wavelength Electronics LFI−3751 temperature controller (excitation wavelength 646 nm, emission acquired from 650 to 800 nm, 20 °C).

### 2.5. Molecular Docking

The three-dimensional structure of the AT11 aptamer was downloaded from the PDB database (https://www.rcsb.org/, accessed on 5 February 2022; PDB entry 2N3M). The dock preparation (DockPrep tool), the assignment of Gasteiger charges and polar hydrogens for G4 nucleic acid were performed using Chimera 1.16. Docking experiments were carried out by the AutoDock 4.2 program using the Lamarckian genetic algorithm (25 runs in an initial population of 150 random individuals, a maximum number of evaluations of 2.5 × 10^6^, rate of mutation and crossover of 0.02 and 0.8 and, finally, elitism value of 1). Using a box (125 × 125 × 125 Å along the x, y, and z axes) with a grid spacing of 0.6 Å, the AT11 structure was centered and left rigid while the ligand was allowed full flexibility. Additionally, the same protocol was applied to understand the interaction between the C_8_ ligand and the GATG repeating unit.

### 2.6. Loading Efficiency Determination

The loading efficiency of the C_8_ ligand by the aptadendrimers was determined, based on the amount of C_8_ entrapped, using Equation (1).

(1) $$\mathrm{Loading}\;\mathrm{efficiency}=\frac{\left[\mathrm{Amount}\;\mathrm{of}\;C_{8}\;\mathrm{added}-\mathrm{Amount}\;\mathrm{of}\;\mathrm{free}\;C_{8}\right]}{\mathrm{Amount}\;\mathrm{of}\;C_{8}\;\mathrm{added}}$$

Briefly, the aptadendrimer (10 μM of aptamer) was incubated with C_8_ (1 μM) under constant stirring using a Hula Mixer Sample Mixer (Thermo Fisher Scientific, Waltham, MA, USA). The measurements of the amount of C_8_ were based on the fluorescence intensity of the ligand in the supernatant and following centrifugation at 3000× *g* for 15 min using a Vivaspin 2 kDa (Sartorius, Gottingen, Germany) in the flowthrough. For fluorescence measurements, a high-precision 3 mm × 3 mm cell (Hellma Analytics, Jena, Germany) was used in a Horiba FluoroMax4 fluorometer (Kyoto, Japan) using suitable excitation and emission wavelengths (492 and 526 nm, respectively). The wavelengths were determined after excitation and emission spectra in a fluorometer.

### 2.7. Drug Release

The C_8_ release profile of aptadendrimer was studied applying the method reported by Carvalho et al. [25]. Using a Slide-A-Lyzer™ MINI Dialysis Device with a 3.5 kDa of molecular cut-off (Thermo Fisher Scientific, USA), 100 μL of aptadendrimer/C_8_ complex was added and dialysis was performed in 1 mL of the final volume of buffer solution under constant stirring using an HulaMixer Sample Mixer (Thermo Fisher Scientific, USA). Samples were collected from the bottom tube (100 μL) along the time window, and buffer was added to the dialysis medium in order to maintain constant volume during the experiment. The released C_8_ content was quantified by measuring the fluorescence of the samples using a high-precision 3 mm × 3 mm cell (Hellma Analytics, Germany) in a Horiba FluoroMax4 fluorometer (Kyoto, Japan) using suitable excitation and emission wavelengths (492 and 526 nm, respectively).

### 2.8. Steady-State Fluorescence Intensity and Anisotropy Measurements

Steady-state fluorescence experiments were performed in a Horiba Jobin Yvon Fluorolog 3-22 (Tokyo, Japan) spectrofluorometer using 0.5 cm × 0.5 cm quartz cuvettes (Hellma Analytics, Germany) at room temperature. The emission spectra of 0.5 µM C_8_ in buffer and upon incubation with increasing aptadendrimer concentrations were recorded with excitation at 488 nm with 2 and 4 nm slits in the excitation and emission, respectively. For fluorescence anisotropy experiments, the samples were excited at 488 nm and the polarized emission was recorded at 520 nm using 5 and 10 nm slits in the excitation and emission, respectively. The steady-state fluorescence anisotropy, 〈r〉, was calculated using Equation (2) as previously described [26]:

(2) $$\langle r\rangle =\frac{I_{VV}-G\cdot I_{VH}}{I_{VV}+2\cdot G\cdot I_{VH}}$$

where $I_{VV}$ and $I_{VH}$ are the intensities of the vertically and horizontally polarized fluorescence emission upon excitation with vertically polarized light, respectively. The G factor ($G=I_{HV}/I_{HH\;}$, with horizontal excitation components) accounts for the monochromator transmission efficiency to the polarization of the light. Data are shown as mean ± standard deviation of 10 measurements.

### 2.9. Time-Resolved Fluorescence Intensity and Anisotropy Measurements

Time-resolved fluorescence experiments were carried out by the single-photon-timing technique as previously described [27,28]. The samples were excited at 488 nm using a BDS-SM-488FBE pulsed picosecond diode laser from Becker & Hickl (Berlin, Germany), and the fluorescence emission was recorded at 520 nm. The fluorescence intensity decays, I(t), were collected with the emission polarizer set at the magical angle regarding the vertically polarized excitation beam. For anisotropy decays, the parallel and perpendicular polarized components of the fluorescence—$I_{VV}\left(t\right)$ and $I_{VH}\left(t\right)$, respectively—to the plane of the excitation beam were alternatively recorded. The instrument response function (IRF) was obtained from the excitation light scattered by a Ludox solution (silica, colloidal water solution, Sigma-Aldrich).

The decays were analyzed using the TRFA Data Processer Advanced (version 1.4) from the Scientific Software Technologies Centre (Belarusian State University, Minsk, Belarus) as previously detailed [27,28]. The reduced χ2 value (<1.3) and the random distribution of weighted residuals/autocorrelation plots were used to evaluate the goodness of the analysis.

Fluorescence intensity decays were analyzed using a sum of discrete exponential terms [26]:

(3) $$I\left(t\right)=\sum_{i=1}^{n}\alpha_{i}\;exp\left(-t/\tau_{i}\right)$$

here $\alpha_{i}$ and $\tau_{i}$ are the pre-exponential (amplitude) and the lifetime of the *i*th decay component of fluorescence, respectively. The amplitude-weighted mean fluorescence lifetime, 〈τ〉, was then calculated according to:

(4) $$\langle \tau \rangle =\sum_{i=1}^{n}\alpha_{i}\;\tau_{i}$$

The fluorescence anisotropy decays, r(t), were analyzed by a sum of discrete exponential terms as previously described [26]:

(5) $$r\left(t\right)=\sum_{i=1}^{n}\beta_{i}\;exp\left(-t/\phi_{i}\right)$$

where $\beta_{i\;}$ and $\phi_{i}\;$ stand for the normalized amplitude and the rotational correlation time of the *i*th anisotropy decay component, respectively.

### 2.10. Cellular Uptake of Aptadendrimers

PNT1A, DU-145, and PC-3 cell lines were seeded in Ibidi 8-well μ-slides (IBIDI, Gräfelfing, Germany) at a final concentration of 1 × 10^5^ cells/well. After 24 h for adhesion, the cells were incubated with aptadendrimers (0.1 μM) and aptadendrimers/C_8_ complexes (0.1 μM and 0.33 μM, respectively) for 1 h. In the uptake mechanism experiment, the cells and all solutions were pre-incubated at 4 °C. Then, they were washed three times with PBS 1×, stained with 1 μM nuclear probe Hoechst 33342 for 15 min, and over again washed with PBS 1×. Images were acquired using a confocal laser scanning microscope (CLSM; Zeiss AxioObserver LSM 710, Carl Zeiss, Oberkochen, Germany) equipped with a plane-apochromatic 63×/DIC objective and lasers (405, 488, 561 and 663 nm). All the images were processed with Zeiss ZEN Black software (Carl Zeiss, Oberkochen, Germany).

### 2.11. Cytotoxic Studies of Aptadendrimers

The cytotoxicity of the aptadendrimer, C_8_-loaded aptadendrimer, AT11 aptamer, and AT11-C_8_ was evaluated in PNT1A, PC-3, and DU-145 cell lines by the MTT assay. The cells were seeded in 96-well plates (2 × 10^4^ cells/mL) and after 24 h for cell adhesion, they were treated with different concentrations of aptadendrimers and complexes for 72 h. In IC_50_ experiments, the C_8_ ligand was successively diluted (from 3.12 μM to 0.024 μM). The wells containing untreated cells were used as control. Then, fresh media containing 3-(4,5-dimethylthiazol-2-yl)-2,5-diphenyltetrazolium bromide salt (MTT; Sigma-Aldrich, St. Louis, MO, USA) was added, followed by further incubation at 37 °C in a humidified atmosphere containing 5% of CO_2_ for 4 h. Formazan crystals were dissolved in DMSO, and their absorbance was recorded at 570 nm using a BioRad ×Mark™ microplate reader (BioRad, Hercules, CA, USA). The cell viability percentages of different treatments were calculated by considering the absorbance of the control as 100% viability. Cell viability was expressed as mean ± SD from at least three different plates with each condition tested in quadruplicate wells. GraphPad Prism 8 (San Diego, CA, USA) was used for data treatment.

## 3. Results and Discussion

For the preparation of the aptadendrimer, we selected a fourth-generation dendrimer of the GATG family, herein referred to as 2[G4]-N_3_ (Figure 1B), previously described by some of us [21]. GATG dendrimers are composed of a gallic acid core, responsible for the multivalency, and long triethylene glycol spacer arms that give flexibility to the dendritic structure. They have been exploited for the preparation of drug [29,30] and gene delivery [31] systems, and the construction of monodisperse nanotools to modulate the complex mechanisms governing multivalent interactions [32,33,34,35]. 2[G4]-N_3_, which is prepared divergently from a triethylene glycol diamine core and the GATG repeating unit (Figure 1A), carries 162 terminal azides that we have used for the bioconjugation of the AT11 aptamer by means of the strain-promoted azide–alkyne cycloaddition (SPAAC) [22,23,24].

With this aim, AT11-NH_2_ (a version of AT11 with a terminal amino group) was first functionalized with a dibenzocyclooctyne (DBCO) using an active ester equipped with a long PEG_13_ linker (DBCO-PEG_13_-NHS, DMSO, 25 °C). The long PEG-based linker was chosen over a long carbon spacer to increase the distance between the dendrimer and the aptamer and consequently, the flexibility of the aptadendrimer (Figure 2A). Additionally, the PEG polymer coating has the advantage improving the biophysical and chemical properties of nanoparticles (such as high hydrophilicity, spatial repulsion, and electrical neutrality) that resulted in higher biocompatibility and blood circulation half-life [36]. Additionally, some therapeutical approaches using PEGylation have already been approved by the Food and Drug Administration of the USA [36]. Despite these positive points, PEG conjugation can present some drawbacks and limitations as adverse side effects caused by the polymer itself or by side products formed during synthesis, unexpected changes in the pharmacokinetic behavior and non-biodegradability of PEG [37]. After purification by dialysis, AT11-PEG_13_-DBCO was obtained in 89% yield. The complete functionalization of AT11 with DBCO was confirmed by UV-Vis (Figure S1) based on their characteristic absorptions at 280 and 309 nm, as described in the Supporting Information.

The subsequent SPAAC bioconjugation of AT11-PEG_13_-DBCO to 2[G4]-N_3_ was performed in DMSO at 37 °C (Figure 2B). Monitoring of the reaction progress was carried out by PAGE until no free aptamer was detected (Figure S2). Several loadings of AT11 aptamer per dendrimer were tested, namely 8, 17, and 34. Since, for the lower loadings, some aptadendrimer aggregation was observed, a loading of 34 was defined for this initial exploration of the aptadendrimer properties and activity. After completion of the AT11 bioconjugation, the aptadendrimer was labelled with a cyanine-5 (Cy5) fluorophore also using a SPAAC. After purification by ultrafiltration (Amicon) to remove any potential unreacted Cy5, 2[G4]-(N_3_)_127_/(AT11)_34_/(Cy5)_1_ was obtained in 97% yield. In this conjugate, a Cy5 loading of 1 was determined by UV-Vis (Figure S3) and checked by fluorescence spectroscopy (Figure S4), as described in the Supporting Information.

Once the synthesis of the aptadendrimer had been performed, we checked the G4 formation of AT11 conjugated on the dendrimer surface by CD spectroscopy (Figure S5). The results showed a characteristic profile of parallel G4 topology (positive and negative bands at 260 and 240 nm, respectively) as previously reported for this aptamer [11]. In addition, it was possible to observe an extra band around 280 nm, a chirality signal that can be explained by the conjugation of AT11 with the PEG_13_ spacer and the dendrimer. This band is attributed to the π-π* transition of the carbonyl groups of the GATG repeating unit that was embedded in a chiral entity becoming CD active [38,39] or can be explained by conjugation with the dendrimer that alters the secondary structure of AT11.

To evaluate if the aptadendrimer can benefit from the enhanced permeability and retention (EPR) effect, we performed a dynamic light scattering (DLS) assay to evaluate its size. The aptadendrimer exhibited a mean particle size of 3.1 nm (Figure S6), which is in accordance with the reference values [40,41,42]. In addition, this nanosize is suitable for cellular internalization (≤100 nm). Additionally, the surface charge of aptadendrimer was measured. A zeta potential of −22.2 mV was revealed for the aptadendrimer, an anionic character with a value in line with others previously described for G4 aptamer-based nanoparticles [43] and the charge adopted by G4 vs. duplex DNA [44]. The negative zeta potential of the aptadendrimer confirms the incorporation of AT11 (highly negative because of the phosphate groups) on the surface of the dendrimer, which is a neutral macromolecule (no charge on the surface of 2[G4]-N3). Not unexpectedly, the data obtained are in agreement with that for Au nanoparticles functionalized with aptamers [43].

Next, we evaluated the binding mode and interactions between the acridine orange ligand C_8_ and AT11 aptamer by molecular docking. C_8_ was chosen not only because of its promising anticancer effects [18,25,45,46], but also because it binds NCL-targeted aptamers and can potentially stabilize G4 structures. A previous report showed that interactions among the C_8_ ligand and G4 structures are mainly end-stacking interactions between the planar acridine moiety and the top and bottom G-quartets [47], resulting in the stabilization of G4 as evidenced by the thermal stability increase [45,46,48,49,50].

**Figure 3:**
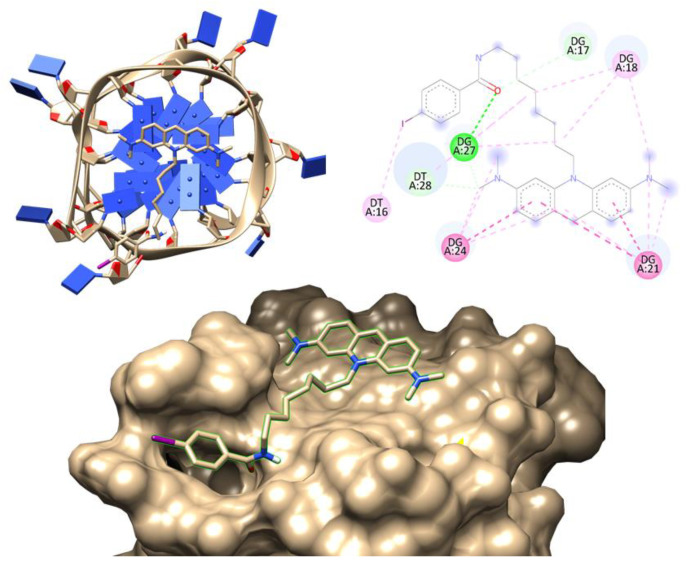
Molecular docking simulation of AT11 aptamer with C_8_. The image depicts the lowest binding free energy complex and the interactions with nucleotide residues.

However, the free C_8_ ligand has a high toxicity in both malignant and normal cell lines [25,43]. For the molecular docking experiments, we used the tridimensional structure of AT11 previously determined by Do et al. [11] and deposited in the Protein Data Bank (PDB entry 2N3M). As expected, the results depicted in Figure 3 showed that C_8_ interacts via π-π stacking with the tetrad (G18-G21-G24; π-π interactions). This can be explained by the planar nature of the acridine moiety that favors these interactions and the protonated nitrogen atoms that are placed near the central carbonyl groups in the G-tetrads.

Additionally, it was observed the interaction of the ligand alkyl linker with the groove (G17-G27-T16-T28). Molecular docking simulations carried out between C_8_ and the GATG repeating unit also confirmed contacts between the acridine scaffold and the gallic acid core of the dendritic structure (Figure S7).

Following this, the aptadendrimer encapsulating C_8_ was prepared and characterized. There were no significant changes in size and zeta potential between the unloaded and C_8_-loaded aptadendrimers, suggesting that the nanoparticle maintained the structure.

To analyze the loading efficiency of the aptadendrimer with C_8_, we incubated C_8_ with the aptadendrimer and the fluorescence of free C_8_ (Figure S8) in the mixture was measured before and after centrifugation (Figure S9) [25]. A percentage of C_8_-loaded aptadendrimer around 98% was revealed through Equation (1).

**Figure 4:**
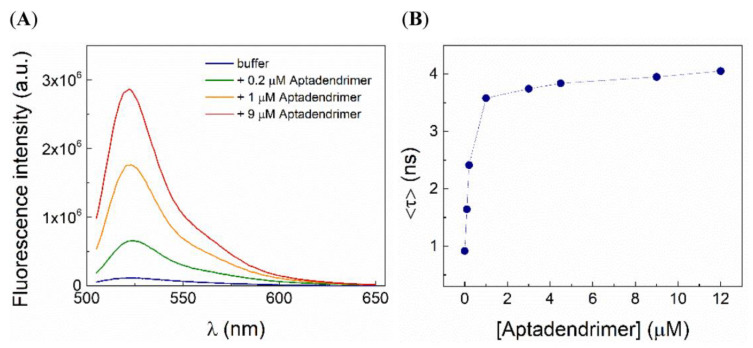
C_8_ fluorescence is severely quenched in the buffer. Variations in (**A**) fluorescence emission spectra and (**B**) the amplitude-weighted mean fluorescence lifetime of 0.5 µM C_8_ with increasing concentrations of aptadendrimer.

The C_8_ association to aptadendrimer was also monitored by both steady-state and time-resolved fluorescence and anisotropy measurements. The variation in the C_8_ fluorescence intensity and the amplitude-weighted mean fluorescence lifetime, 〈τ〉, upon complexation with aptadendrimers are shown in Figure 4A,B, respectively. Notably, the fluorescence intensity increased more than 20-fold and the 〈τ〉 enhanced from 0.86 ns in the buffer to 4.05 ns upon C_8_ binding to the aptadendrimers, supporting that C_8_ fluorescence in the buffer is severely quenched. Considering the C_8_ structure (Figure 1D), the iodine atom acts as an effective intramolecular quencher of the conjugated dye in an aqueous solution due to the heavy atom effect; upon its binding to the aptadendrimer, C_8_ must adopt a more extended conformation that prevents the iodine quencher effect.

**Figure 5:**
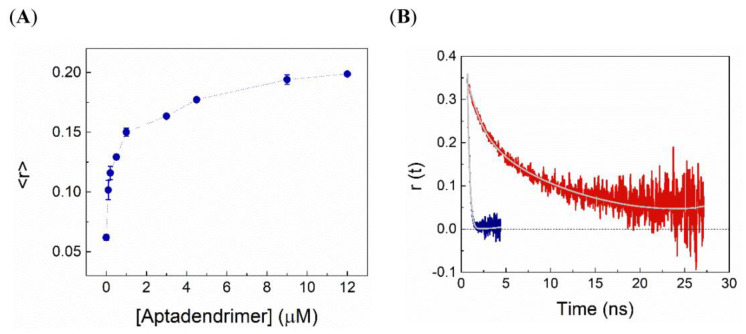
C_8_ binding to the aptadendrimer. (**A**) Changes in the steady-state fluorescence anisotropy of 0.5 µM C_8_ with increasing aptadendrimer concentrations. (**B**) Fluorescence anisotropy decays of 0.5 µM C_8_ in buffer (blue) and in the presence of 9 µM of aptadendrimer (red). The rotational correlation times increased upon complexation denoting the changes in the segmental motion of bound C_8_ ($\phi_{1}$ from ~0.01 to 1.5 ns) and it is slower global tumbling upon binding to the large dendrimers ($\phi_{2}$ increased from 0.15 to 10–12 ns).

To further confirm the C_8_-aptadendrimer complex formation, fluorescence anisotropy experiments were also carried out. A relatively high steady-state fluorescence anisotropy in buffer was obtained for a small fluorophore (〈*r*〉 = 0.062 + 0.006), which is explained in part by the short fluorescence lifetime of C_8_ in an aqueous solution. Remarkably, upon increasing the aptadendrimer concentration, the anisotropy of C_8_ steadily augmented until attaining a plateau at 〈*r*〉~0.20 + 0.001 (Figure 5A). These data reveal that the hydrodynamic volume of C_8_ increased in the presence of the aptadendrimer, confirming its complexation and the slowing down of the overall rotational tumbling of the complex during its fluorescence lifetime. Moreover, time-resolved fluorescence anisotropy experiments further confirmed the C_8_ complexation. Indeed, the fluorescence anisotropy decay of C_8_ was greatly affected by the addition of the aptadendrimer (Figure 5B). First, the fast rotational correlation time ($\phi_{1}$) of C_8_ was enhanced upon complexation towards 1.4 ns, which reflects the segmental motion of C_8_ bound to the PEG_13_-AT11 in the dendrimer. Moreover, the longer rotational correlation time ($\phi_{2}$), assigned to the overall rotational motion, increased significantly from 0.14 ns for C_8_ in solution to 10–12 ns upon binding to the aptadendrimer. Altogether, our results clearly show that C_8_ binds to the aptadendrimer.

**Figure 6:**
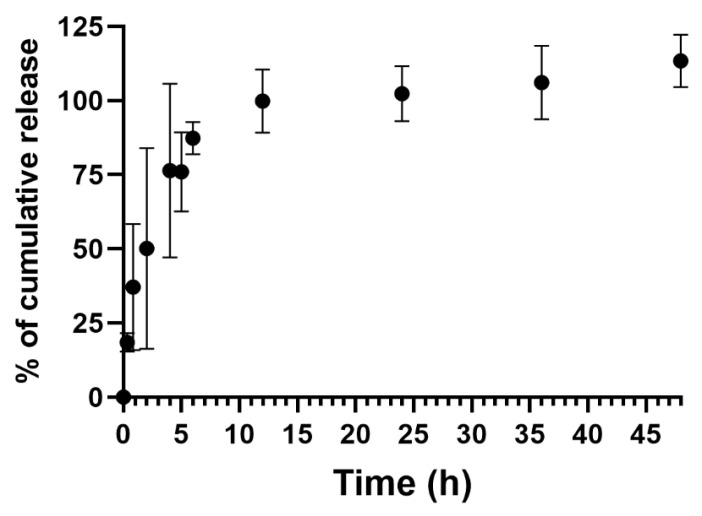
Cumulative release profile of C_8_ from the C_8_–aptadendrimer complex. The experiment was performed in 20 mM KPi (pH = 6.9) supplemented with 65 mM KCl for 48 h (*n* = 3).

The release profile of C_8_ from the aptadendrimer complex (Figure 6) was evaluated in 20 mM KPi (pH = 6.9) supplemented with 65 mM KCl for 48 h, with samples collected at different time points. The results evidenced that the release is fast and most noticeable after 4 h and it has a maximum cumulative release profile at 12 h.

**Figure 7:**
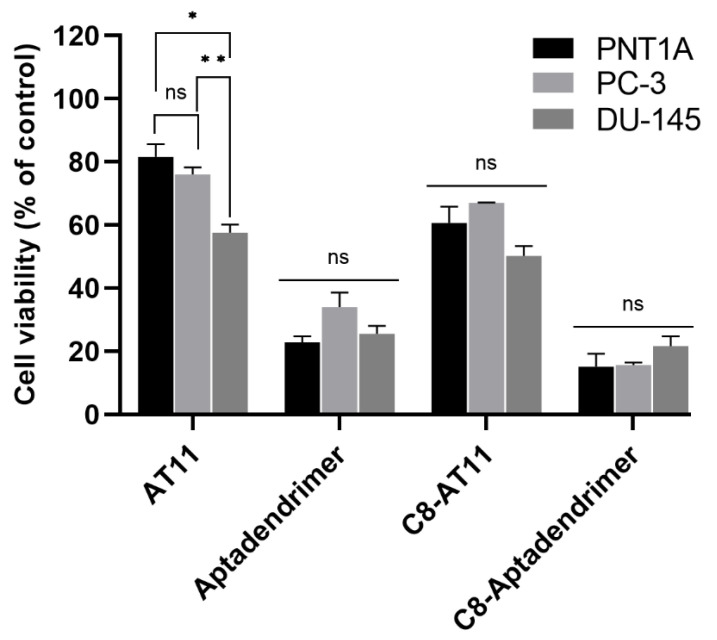
Histograms of cell viability of PNT1A, PC-3 and DU-145 cells after incubation with AT11 (6.6 µM), C_8_-AT11 (6.6 µM AT11 + 0.66 µM C_8_), aptadendrimer (0.2 µM) and C_8_-aptadendrimer (0.2 µM aptadendrimer + 0.66 µM C_8_) for 3 days. Results are presented in percentage normalized regarding control. Statistical analysis (One-way ANOVA) was performed using GraphPad Prism software: * *p* < 0.05; ** *p* < 0.01; ns—not significant (*p* > 0.05). Complementary statistical analysis among all conditions in different cell lines are presented in Table S1.

Next, we performed in vitro studies to access the cellular internalization and viability of the aptadendrimer in two prostate cancer cell lines (PC-3 and DU-145) and one non-malignant cell line (PNT1A). The cell viability was determined by the MTT assay after incubation of the AT11, C_8_-AT11 complex, aptadendrimer, and C_8_-aptadendrimer complex for 3 days. The results are presented in Figure 7.

Free AT11 elicited only a minor effect on the viability of both PC-3 and PNT1A cells (80% of mean viability) and 58% on the viability of DU-145 cells, in which DU-145 and PC-3 were both androgen-independent with moderate and high metastatic potential, respectively [51]. On the other hand, free C_8_ reduced the viability of all cell lines (Figure S10), showing a non-selective cytotoxic effect, as previously seen [43,49].

The aptadendrimer presented higher cytotoxicity than free AT11 in all cell lines. Indeed, incubation of the aptadendrimer (at the same concentration of AT11 as above) for 3 days dramatically increased its toxicity by 3.56 times for PNT1A, and 2.23 and 2.25 times for PC-3 and DU-145 cells (ratios among the viabilities of free AT11 and aptadendrimer), respectively (Figure 7). The cell viability decreased to *ca.* 23% for PNT1A, 34% for PC-3, and 26% for DU-145 cells. Regarding C_8_–aptadendrimer, after 3 days, it caused a ca. 15%, 16%, and 22% reduction in viability of PNT1A, PC-3, and DU-145 cells, respectively. The similar toxicity for both cancer and non-malignant cells points to a lack of selectivity for the aptadendrimer to deliver C_8_ to the target cancer cells.

In order to evaluate the uptake, we treated the cancerous and the non-malignant cell lines with the aptadendrimer and C_8_–aptadendrimer complex labelled with Cy5 and recorded images using a confocal laser scanning microscope (CLSM). Firstly, we performed a live imaging of the aptadendrimer over 1 h in PC-3 and PNT1A cells (Figures S11 and S12). The results evidenced a fast internalization of the aptadendrimer. It was not possible to observe colocalization of the aptadendrimer with an NCL antibody in both cell lines, suggesting that internalization is not actively mediated by cell surface NCL. In a previous report, we showed that free Cy5-AT11 and C_8_-AT11 were able to colocalize NCL in HeLa cancer cells and that the complex remained stable during cell trafficking, while free C_8_ localized with nucleoli [18]. To clarify the cellular uptake mechanism of the aptadendrimer, we incubated the cells with aptadendrimer at a low temperature (4 °C). At lower temperatures, cells reduced their metabolism, and the membrane increased rigidity and blocked energy-dependent uptake and passive diffusion [52]. The passive diffusion mechanism is predominantly limited to small, uncharged molecules that travel down concentration gradients. Our results (Figure S13) evidenced the absence of an aptadendrimer fluorescence signal for both cell lines at low temperatures. Of note, incubation at 4 °C also influenced Hoechst migration and, consequently, the labeling of nuclei. This result demonstrates that the uptake mechanism is not based on passive transport but rather on another pathway. Usually, the cellular uptake of nanocarriers requires endocytosis [53]. In the specific case of dendrimer nanocarriers, it is extensively described that internalization can occur through different endocytic routes [54,55,56,57,58]. We propose that in this case, internalization proceeds via endocytosis through an NCL-independent way. We can infer that the uptake mechanism can be intrinsically related to the high toxicity of aptadendrimers in both cell lines. Previous studies suggested that endocytosis pathways can be identified by the combination of different inhibitors, endocytic markers and genetic approaches [59]. Nevertheless, there is a possibility of side effects, upregulation of compensatory mechanisms and their effect can vary between different cell lines [59].

**Figure 8:**
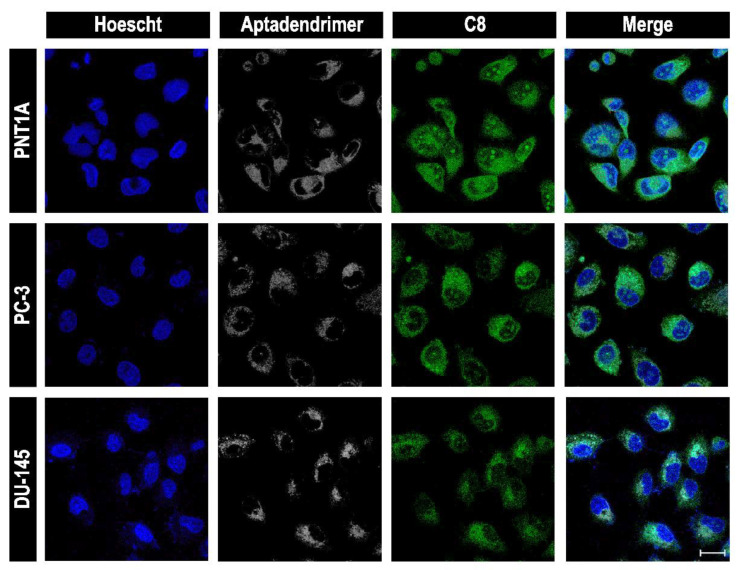
Confocal laser scanning microscopy of prostate cells (PNT1A, PC-3, and DU-145) incubated 1 h with the C_8_–aptadendrimer complex. Results evidenced that the aptadendrimer and C_8_–aptadendrimer complex is able to enter into all cell lines. Additionally, colocalization among the aptadendrimer and C8 was observed which demonstrates the formation of a complex *in cellulo.* Cy5-labelled aptadendrimer is represented as a white signal and the C_8_ ligand as green fluorescence. Cell nuclei are stained with Hoechst 33,342 (blue signal). Scale bar: 20 µm.

Subsequently, whether the conjugation of the C_8_ ligand with the aptadendrimer influences the internalization process was analyzed. For this, cell lines were incubated for 1 h with the C_8_–aptadendrimer complex and after, imaged by CLSM. According to Figure 8, it is possible to claim that the aptadendrimer internalizes differently than free AT11 and that it is localized in the cytoplasm of the cells, demonstrating that the association of C_8_ to the aptadendrimer did not affect the internalization and uptake into the cells.

Considering that the lysosomal escape of nanoparticles is a crucial parameter for an efficient intracellular delivery [60], we decided to analyze the capacity of the developed aptadendrimer to escape from the lysosomal compartmentalization in PC-3 and PNT1A cells. From CLSM and using the LysoView 540 probe to stain lysosomes, we observed that PC-3 cells seemed to easily internalize the aptadendrimer and presented a higher fluorescence intensity than PNT1A (Figure S14). Regarding colocalization coefficients (Table S2), they were similar and relatively high, which could indicate that the aptadendrimer was unable to escape lysosome degradation in both cell lines. Although, the free aptadendrimer that did not colocalize with LysoView 540 (in both cell lines) could influence the similar results in the cell viability after aptadendrimer treatment. The colocalization of the aptadendrimer with the lysosomal marker corroborates the previous experiments where we confirmed that the passive diffusion mechanism is not the uptake route for aptadendrimer internalization. A recent study conducted by Moreno-Echeverr et al. summarizes a detailed experimental protocol for sample preparation, staining and imaging to improve the reproducibility of nanoparticles’ colocalization with lysosomes [61].

## 4. Conclusions

This study reports the synthesis of an aptadendrimer, a fourth-generation GATG dendrimer functionalized on the surface with 34 copies of the AT11 aptamer loading the acridine orange ligand C_8_. The aptamer AT11 adopts a G4 structure confirmed by CD spectroscopy before and after conjugation with the dendrimer. The aptadendrimer was prepared by conjugation via SPAAC, leading to nanostructures with a particle size of 3.1 nm suitable for in vitro studies. The association of the aptadendrimer with C_8_, an acridine orange ligand with anticancer properties that binds/stabilizes the G4 structure of AT11, was confirmed by steady-state and time-resolved fluorescence and anisotropy measurements. Fluorimetry experiments also showed a high loading capacity of the aptadendrimer with C_8_. The release of C_8_ from the aptadendrimer was significant after 4 h and showed a maximum cumulative release profile after 12 h. Confocal microscopy indicated that the C_8_–aptadendrimer complex was efficiently taken up into the cells and it was maintained during cell internalization and trafficking. The internalization of the aptadendrimer or complex with C_8_ was not mediated by NCL or passive diffusion but by other internalization pathways such as endocytosis. The compartmentalization of aptadendrimers in the lysosomes was also verified. Cell viability studies showed that the aptadendrimer and C_8_ complex presented a non-selective cytotoxic effect.

## Data Availability

The data presented in this study are available on request from the corresponding author.

## Supplementary Materials

The following supporting information can be downloaded at: https://www.mdpi.com/article/10.3390/pharmaceutics14112456/s1, Figure S1: UV-Vis spectrum of AT11-PEG_13_-DBCO in 20 mM potassium phosphate pH 6.9, 65 mM KCl; Figure S2: Monitoring of the SPAAC progression by PAGE (15%, 150 V, TAE running buffer, staining with EtBr) confirmed complete reaction. SPAAC reaction (R) and a calibration ladder that accounts for 100, 50, 25, 12.5, 5 and 1% of the AT11-PEG_13_-DBCO added to the reaction; Figure S3: UV-Vis spectrum of aptadendrimer 2[G4]-(N_3_)_127_/(AT11)_34_/(Cy5)_1_ in 20 mM potassium phosphate pH 6.9, 65 mM KCl; Figure S4: Fluorescence spectrum aptadendrimer 2[G4]-(N_3_)_127_/(AT11)_34_/(Cy5)_1_ in 20 mM potassium phosphate pH 6.9, 65 mM KCl (λ_exc._ 646 nm); Figure S5: CD spectrum of aptadendrimer 2[G4]-(N_3_)_127_/(AT11)_34_/(Cy5)_1_ in 20 mM potassium phosphate pH 6.9, 65 mM KCl; Figure S6: DLS of aptadendrimer (1 mg/mL) measured at 25 °C in KPi buffer supplemented with 65 mM of KCl; Figure S7: Molecular docking simulation of C_8_ ligand with GATG repeating unit; Figure S8: Excitation and emission fluorescence spectra of C_8_; Figure S9: Loading efficiency experiment measured by the fluorescence spectrum of C_8_ before and after centrifugation; Figure S10: Cell viability histograms and dose-response data of cells after C_8_ incubation for 3 days analyzed by MTT assay in PNT1A, DU-145, and PC-3; Figure S11: Aptadendrimer uptake in PC-3 cells at different times assessed by confocal laser scanning microscopy (CLSM). The white signal fluorescence represents the Cy5 labelled aptadendrimer. Cell nuclei (blue signal) are stained with Hoechst 33342; Figure S12: Cellular uptake of the aptadendrimer in PNT1A cells along time. Cy5 labelled aptadendrimer is represented with a white signal fluorescence and cell nuclei are stained with Hoechst 33342 (blue signal); Figure S13: Cellular uptake of the aptadendrimer in PNT1A and PC-3 cell lines when incubated at low temperatures (4 °C). Cell nuclei are stained with Hoechst 33342 (blue signal); Figure S14: CLSM of lysosomal compartmentalization experiment in PC-3 and PNT1A cell lines. Cy5 labelled aptadendrimer is represented with a white signal fluorescence, lysosomes stained with LysoView 540 probe (green signal), and cell nuclei are stained with Hoechst 33342 (blue signal); Table S1: ANOVA analysis of experiments conducted in healthy and cancerous cell lines in distinct conditions; Table S2: Colocalization coefficients of lysosomal compartmentalization experiment in PC-3 and PNT1A cell lines. Reference [62] is cited in supplementary materials.
