# Supplementary material for: Gallic Acid–Triethylene Glycol Aptadendrimers Synthesis, Biophysical Characterization and Cellular Evaluation
Source: Pharmaceutics. 2022 Nov 14;14(11):2456. doi: 10.3390/pharmaceutics14112456 (PMC9696068; doi:10.3390/pharmaceutics14112456)
Supplement: Supplementary file 1 [file pharmaceutics-14-02456-s001.zip › pharmaceutics-1969602-supplementary.pdf]

Gallic Acid–Triethylene Glycol Aptadendrimer Synthesis, Biophysical Characterization and Cellular Evaluation

André Miranda [1], Roi Lopez-Blanco [2], Jéssica Lopes-Nunes [1], Ana M. Melo [3,4], Maria Paula Cabral Campello [5,6], António Paulo [5,6], Maria Cristina Oliveira [5,6], Jean-Louis Mergny [7], Paula A. Oliveira [8], Eduardo Fernandez-Megia [2,*] and Carla Cruz [1,9,*]

1 CICS-UBI—Centro de Investigação em Ciências da Saúde, Universidade da Beira Interior, Av. Infante D. Henrique, 6201-506 Covilhã, Portugal
2 Centro Singular de Investigación en Química Biolóxica e Materiais Moleculares (CIQUS), Departamento de Química Orgánica, Universidade de Santiago de Compostela, Jenaro de la Fuente s/n, 15782 Santiago de Compostela, Spain
3 iBB—Institute for Bioengineering and Biosciences, Instituto Superior Técnico, Universidade de Lisboa, Av. Rovisco Pais, 1049-001 Lisboa, Portugal
4 Associate Laboratory i4HB—Institute for Health and Bioeconomy at Instituto Superior Técnico, Universidade de Lisboa, Av. Rovisco Pais, 1049-001 Lisboa, Portugal
5 Centro de Ciências e Tecnologias Nucleares, Instituto Superior Técnico, Universidade de Lisboa, Estrada Nacional 10 (km 139.7), 2695-066 Bobadela LRS, Portugal
6 Departamento de Engenharia e Ciências Nucleares, Instituto Superior Técnico, Universidade de Lisboa, Estrada Nacional 10 (km 139.7), 2695-066 Bobadela LRS, Portugal
7 Laboratoire d'Optique et Biosciences, Ecole Polytechnique, CNRS, INSERM, Institut Polytechnique de Paris, 91128 Palaiseau, France
8 Centre for Research and Technology of Agro-Environmental and Biological Sciences (CITAB), Inov4Agro, University of Trás-os-Montes and Alto Douro (UTAD), Quinta de Prados, 5000-801 Vila Real, Portugal
9 Departamento de Química, Universidade da Beira Interior, Rua Marquês de Ávila e Bolama, 6201-001 Covilhã, Portugal
* Correspondence: ef.megia@usc.es (E.F.-M.); carlacruz@fcsaude.ubi.pt (C.C.)

Aptadendrimer synthesis: surface functionalization of 2[G4]-N_3 with AT11 aptamer and fluorophore labelling

AT11-PEG_{13}-DBCO

DBCO-PEG_{13}-NHS (5.34 mg, 5.10 μmol, 25 equiv) was added to a solution of AT11-NH_2 (1.84 mg, 0.20 μmol) in DMSO (75 μL) and Et_3N (0.1 μL, 0.61 μmol) under Ar. After 24 h of stirring at room temperature, the reaction mixture was purified by dialysis (1 × 500 mL H_2O, 3 × 500 mL 20 mM potassium phosphate pH 6.9, 65 mM KCl, 3 × 500 mL H_2O; MWCO 1 KDa, Spectra/Por 6) to afford AT11-PEG_{13}-DBCO (1.82 mg, 89%).

The number of DBCO per AT11 ($N_{DBCO/AT11}$) was determined by UV-Vis (**Figure S1**) using the absorbance of AT11-PEG_{13}-DBCO at 280 nm (due to AT11 and DBCO) and 309 nm (due to DBCO). The calculation was done using the following equations:

$$[\mathrm{DBCO}] = \frac{A^{309}_{\mathrm{DBCO}}}{\varepsilon^{309}_{\mathrm{DBCO}}} \qquad \textbf{(S1)}$$

$$A^{260}_{\mathrm{AT11}} = 2A^{280}_{\mathrm{AT11}} \qquad \textbf{(S2)}$$

$$A^{280}_{\mathrm{AT11}} = A^{280}_{\mathrm{Total}} - (1.089 \times A^{309}_{\mathrm{DBCO}}) \qquad \textbf{(S3)}$$

$$[\mathrm{AT11}] = \frac{A^{260}_{\mathrm{AT11}}}{\varepsilon^{260}_{\mathrm{AT11}}} = \frac{2A^{280}_{\mathrm{AT11}}}{\varepsilon^{260}_{\mathrm{AT11}}} = \frac{2\left(A^{280}_{\mathrm{Total}} - \left(1.089 \times A^{309}_{\mathrm{DBCO}}\right)\right)}{\varepsilon^{260}_{\mathrm{AT11}}} \qquad \textbf{(S4)}$$

$$N_{\mathrm{DBCO/AT11}} = \frac{[\mathrm{DBCO}]}{[\mathrm{AT11}]} \qquad \textbf{(S5)}$$

where $\varepsilon^{309}_{\mathrm{DBCO}}$ is the extinction coefficient of DBCO at 309 nm (12000 $M^{-1}cm^{-1}$) and $\varepsilon^{260}_{\mathrm{AT11}}$ the extinction coefficient of AT11 at 260 nm (264800 $M^{-1}cm^{-1}$).

Considering the known absorbance ratios of AT11 at 260 and 280 nm (Equation (S2)) and of DBCO at 280 and 309 nm (Equation (S3)) [1], a $N_{DBCO/AT11}$ of 1 was determined from their molar concentrations (Equation (S5)).

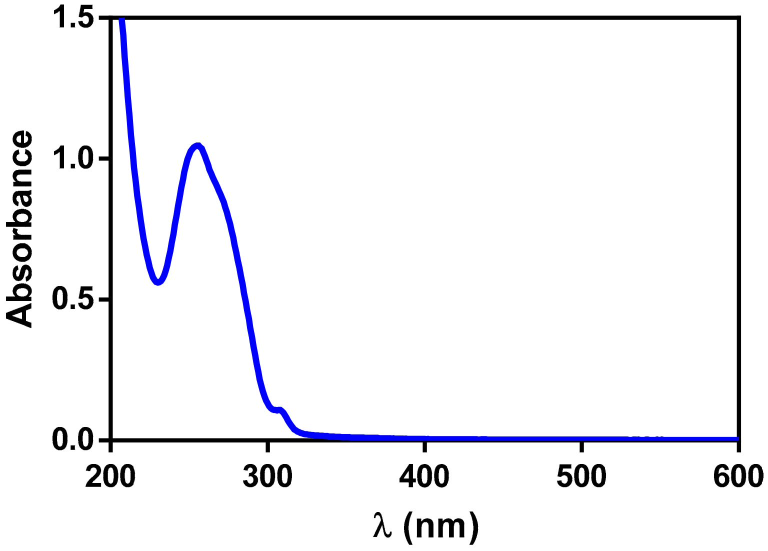

Figure S1. UV-Vis spectrum of AT11-PEG$_{13}$-DBCO in 20 mM potassium phosphate pH 6.9, 65 mM KCl.

2[G4]-(N_3)$_{127}$/(AT11)$_{34}$/(Cy5)$_1$

A solution of AT11-PEG$_{13}$-DBCO (1.68 mg, 0.17 µmol, 34 equiv) in DMSO (1.4 mL) was added to a solution of 2[G4]-N_3 (0.24 mg, 4.96 nmol) in DMSO (1.2 mL). After 24 h of stirring at 37 ºC, LiBr (91.8 mg, 1.06 mmol, equivalent to 400 mM) was added and stirring continued for 96 h till no free AT11-PEG$_{13}$-DBCO was detected by PAGE (15%, 150 V, TAE running buffer, staining with EtBr; **Figure S2**). Then, Cy5-DBCO (4.61 µg, 5.03 nmol, 1 equiv) was added. The reaction mixture was stirred at room temperature for 24 h protected from light and then, purified by Amicon (3 × 5 mL H_2O, 4 × 5 mL 20 mM potassium phosphate pH 6.9, 65 mM KCl; MWCO 3 KDa) to afford 2[G4]-(N_3)$_{127}$/(AT11)$_{34}$/(Cy5)$_1$ (1.85 mg, 97%). A Cy5 loading of 1 was determined by UV-Vis (**Figure S3**) using an extinction coefficient of 250000 $M^{-1}cm^{-1}$ for Cy5 at 646 nm.

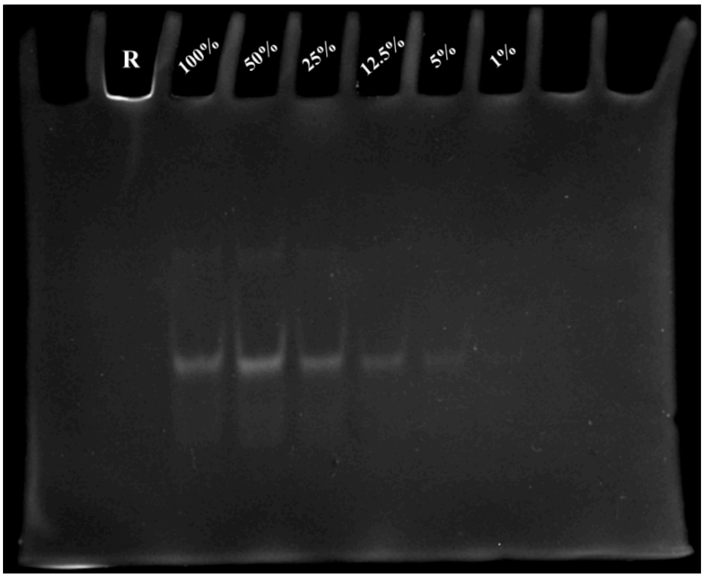

Figure S2. Monitoring of the SPAAC progression by PAGE (15%, 150 V, TAE running buffer, staining with EtBr) confirmed complete reaction. SPAAC reaction (R) and a calibration ladder that accounts for 100, 50, 25, 12.5, 5 and 1% of the AT11-PEG$_{13}$-DBCO added to the reaction.

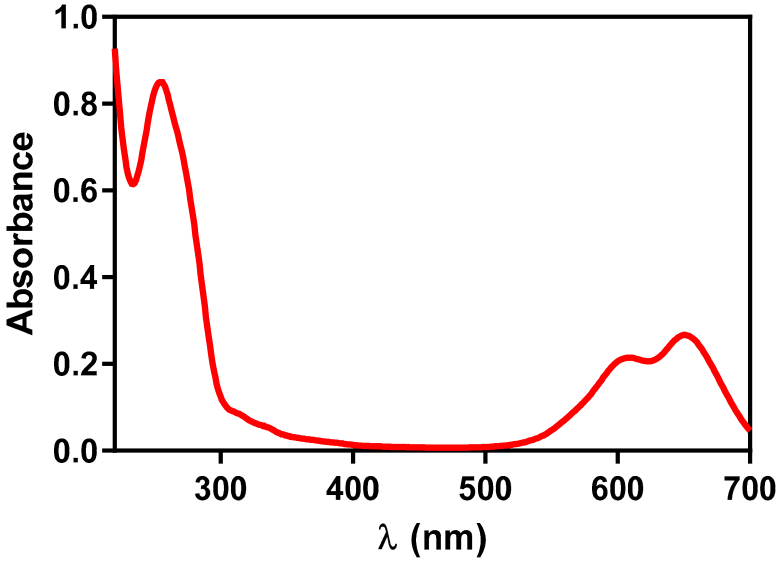

Figure S3. UV-Vis spectrum of aptadendrimer 2[G4]-$(N_3)_{127}$/$(AT11)_{34}$/$(Cy5)_1$ in 20 mM potassium phosphate pH 6.9, 65 mM KCl.

Additional characterization data

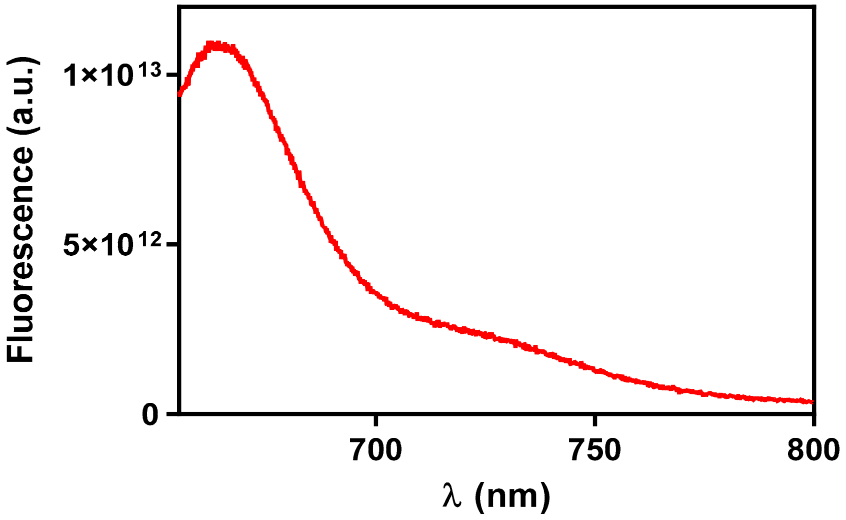

Figure S4. Fluorescence spectrum aptadendrimer 2[G4]-$(N_3)_{127}$/$(AT11)_{34}$/$(Cy5)_1$ in 20 mM potassium phosphate pH 6.9, 65 mM KCl ($\lambda_{exc.}$ 646 nm).

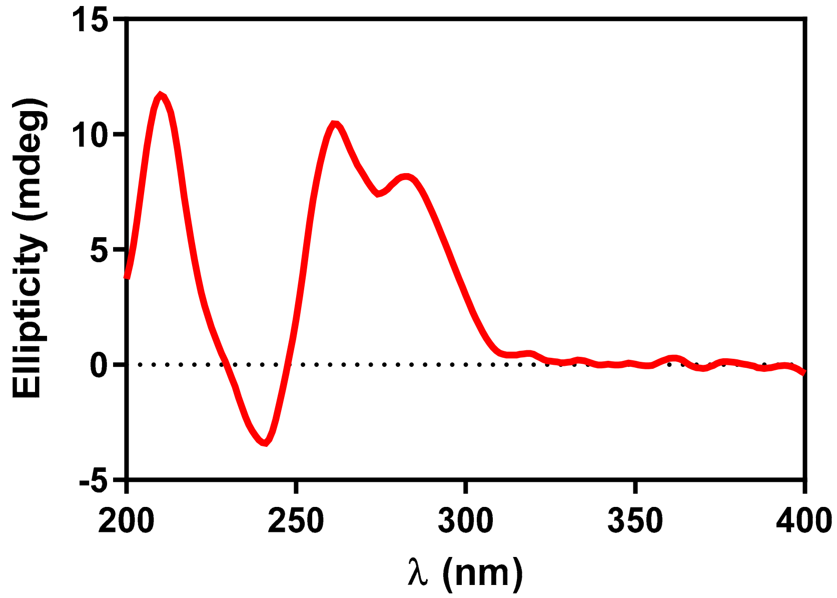

Figure S5. CD spectrum of aptadendrimer 2[G4]-$(N_3)_{127}$/$(AT11)_{34}$/$(Cy5)_1$ in 20 mM potassium phosphate pH 6.9, 65 mM KCl.

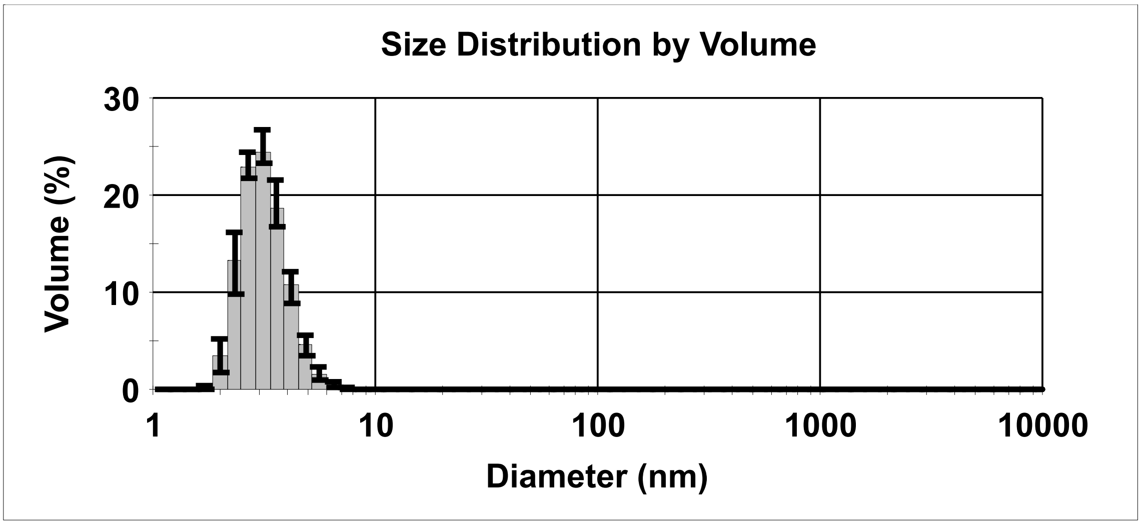

Figure S6. DLS of aptadendrimer (1 mg/mL) measured at 25 ºC in KPi buffer supplemented with 65 mM of KCl.

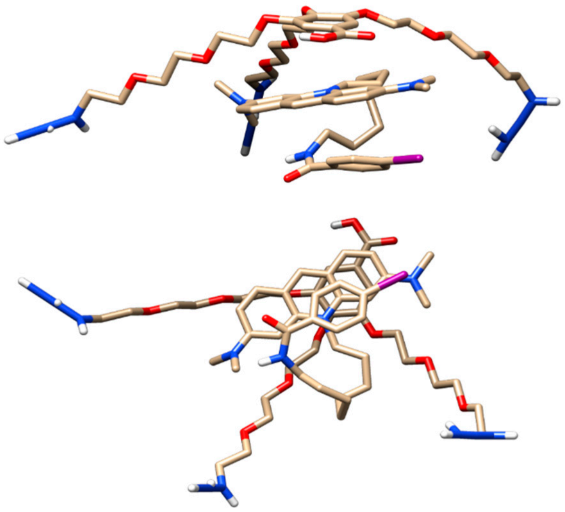

Figure S7. Molecular docking simulation of C_8 ligand with GATG repeating unit.

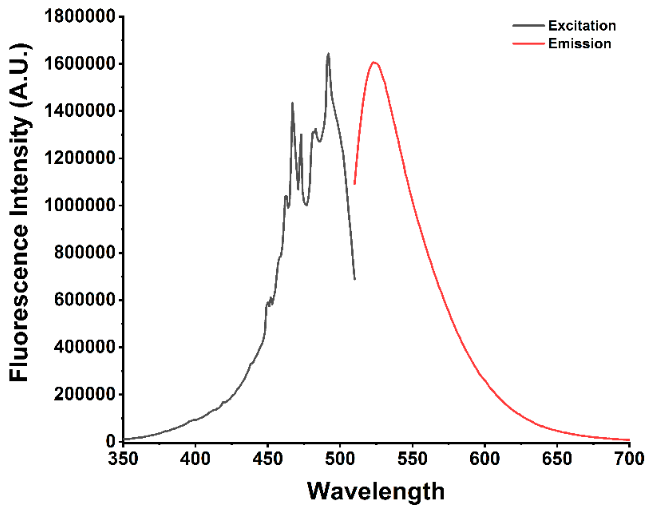

Figure S8. Excitation and emission fluorescence spectra of C_8.

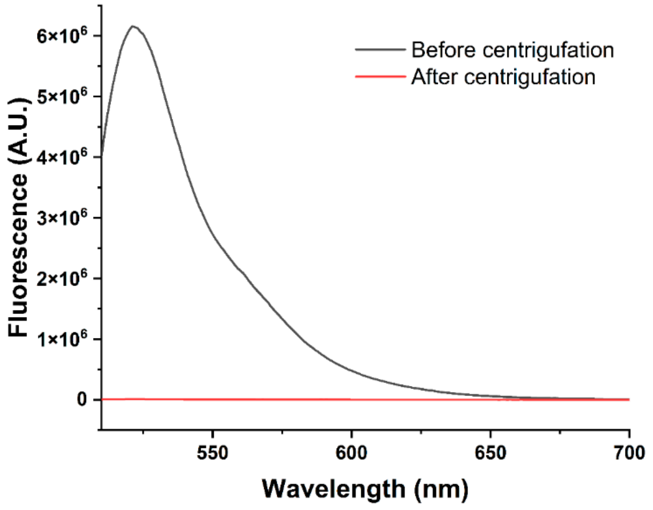

Figure S9. Loading efficiency experiment measured by the fluorescence spectrum of C_8 before and after centrifugation.

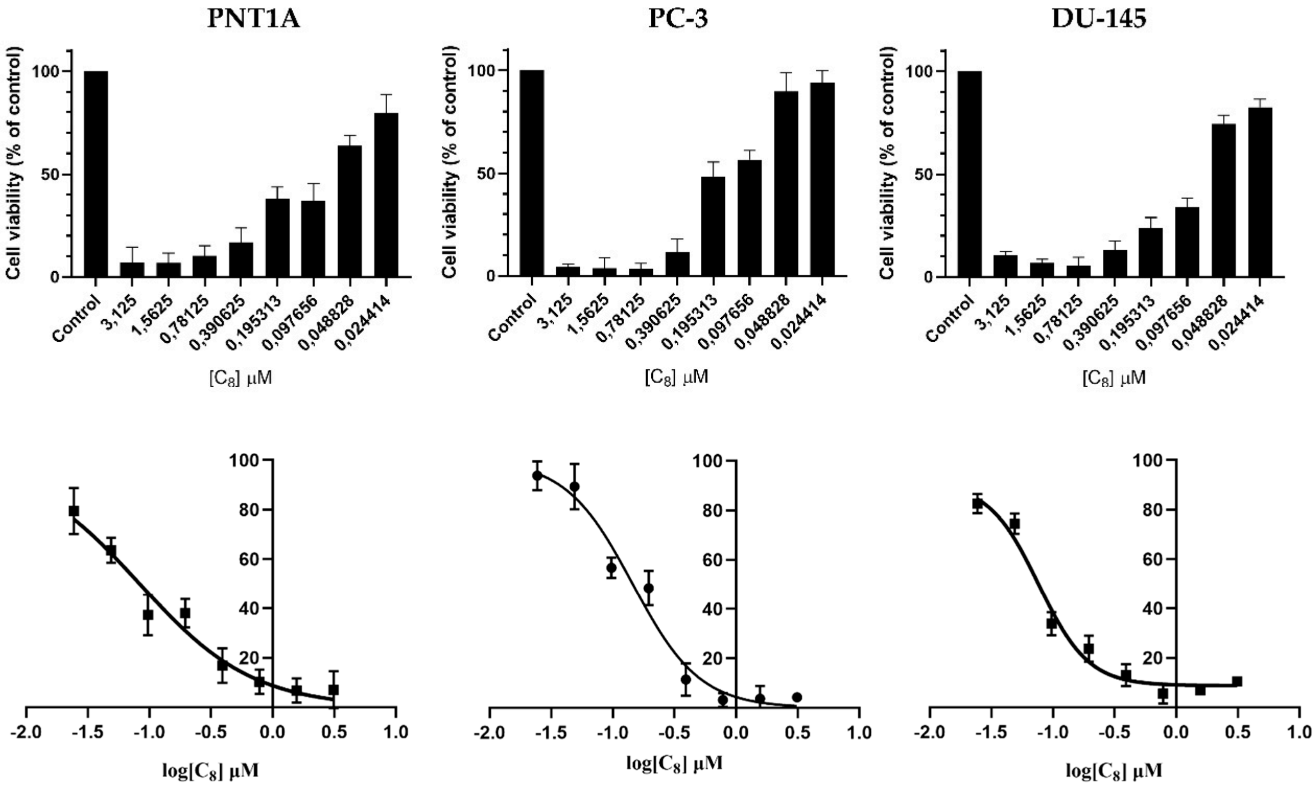

Figure S10. Cell viability histograms and dose-response data of cells after C_8 incubation for 3 days analyzed by MTT assay in PNT1A, DU-145, and PC-3.

Table S1. ANOVA analysis of experiments conducted in healthy and cancerous cell lines in distinct conditions.

**Cell Line**	**PNT1A**	**PC-3**	**DU-145**
Control *vs* AT11	*	***	****
Control *vs* C8-AT11	****	****	****
Control *vs* Aptadendrimer	****	****	****
Control *vs* C8-Aptadendrimer	****	****	****
AT11 *vs* C8-AT11	*	ns	ns
AT11 *vs* Aptadendrimer	****	****	****
AT11 *vs* C8-Aptadendrimer	****	****	****
C8-AT11 *vs* Aptadendrimer	****	****	****
C8-AT11 *vs* C8-Aptadendrimer	****	****	****
Aptadendrimer *vs* C8-Aptadendrimer	ns	**	ns

Statistical analysis (One-way ANOVA) was performed using GraphPad Prism software: *$p < 0.05$; ** $p < 0.01$; *** $p < 0.001$; **** $p < 0.0001$; ns – not significant ($p > 0.05$).

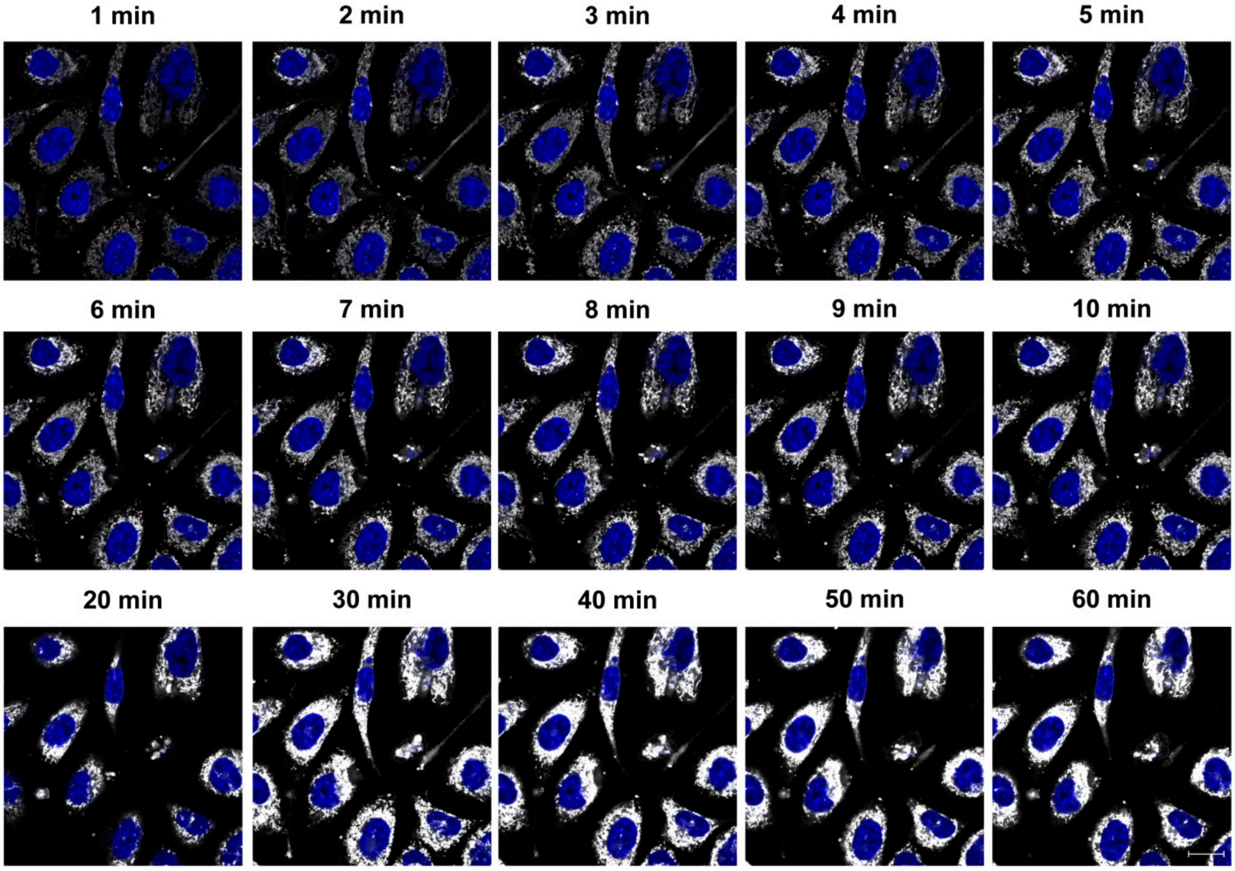

Figure S11. Aptadendrimer uptake in PC-3 cells at different times assessed by confocal laser scanning microscopy (CLSM). The white signal fluorescence represents the Cy5 labelled aptadendrimer. Cell nuclei (blue signal) are stained with Hoechst 33342. Scale bar: 20 µm.

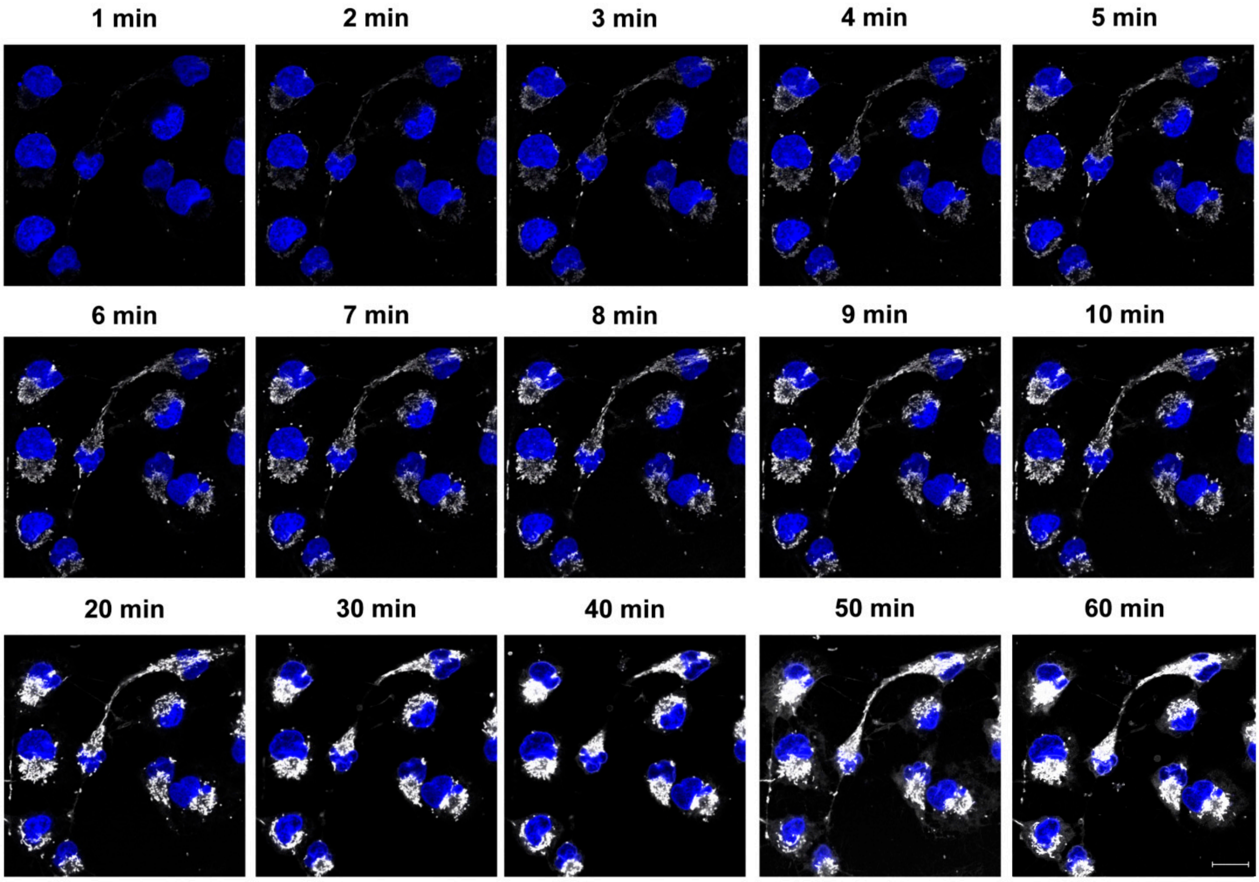

Figure S12. Cellular uptake of the aptadendrimer in PNT1A cells along time. Cy5 labelled aptadendrimer is represented with a white signal fluorescence and cell nuclei are stained with Hoechst 33342 (blue signal). Scale bar: 20 µm.

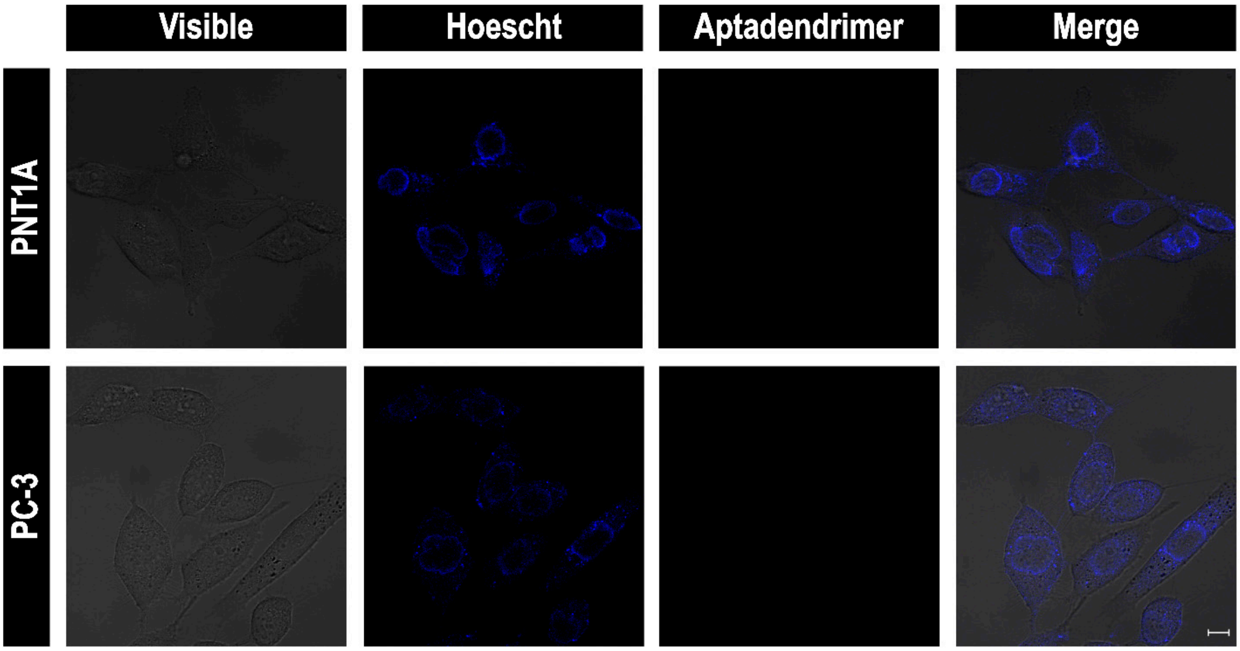

Figure S13. Cellular uptake of the aptadendrimer in PNT1A and PC-3 cell lines when incubated at low temperatures (4 ºC). Cell nuclei are stained with Hoechst 33342 (blue signal). Scale bar: 20 μm.

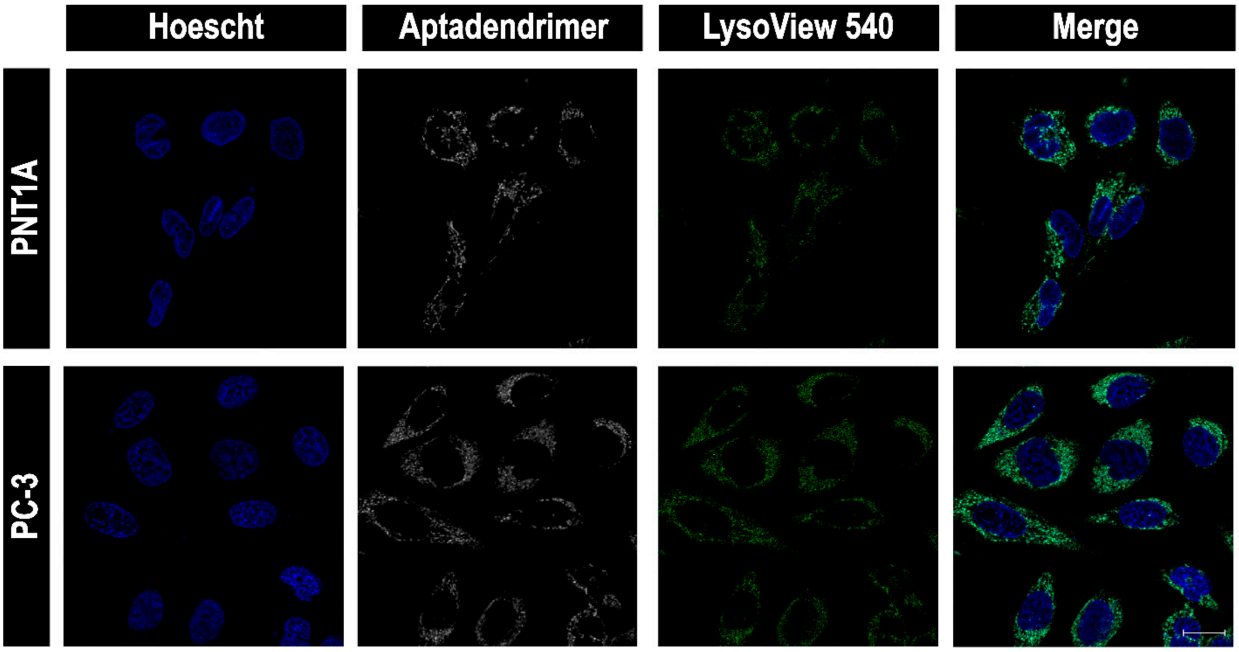

Figure S14. CLSM of lysosomal compartmentalization experiment in PC-3 and PNT1A cell lines. Cy5 labelled aptadendrimer is represented with a white signal fluorescence, lysosomes stained with LysoView 540 probe (green signal), and cell nuclei are stained with Hoechst 33342 (blue signal). Scale bar: 20 µm.

Table S2. Colocalization coefficients of lysosomal compartmentalization experiment in PC-3 and PNT1A cell lines.

	**Mean Fluorescence Intensity**		**Colocalization Analysis**		
	Aptadendrimer	LysoView	Pearson's Coefficient	Mander's Coefficient M1	Mander's Coefficient M2
**PC-3**	6.188 ± 2.583	3.284 ± 1.312	0.7910 ± 0.0193	0.7850 ± 0.0386	0.6350 ± 0.0156
**PNT1A**	3.549 ± 0.627	1.880 ± 0.268	0.7477 ± 0.0119	0.6090 ± 0.0313	0.6800 ± 0.0171

M1 - fraction of LysoView overlapping Aptadendrimer | M2- fraction of Aptadendrimer overlapping LysoView

Reference

1. Liu, D.; Yang, J.; Wang, H.F.; Wang, Z.; Huang, X.; Wang, Z.; Niu, G.; Hight Walker, A.R.; Chen, X. Glucose Oxidase-Catalyzed Growth of Gold Nanoparticles Enables Quantitative Detection of Attomolar Cancer Biomarkers. Anal. Chem. 2014, 86, 5800–5806, https://doi.org/10.1021/ac500478g.
